# Discovery and Validation of Potential Serum Biomarkers for Heart Failure by Untargeted Metabolomics

**DOI:** 10.1155/2024/7004371

**Published:** 2024-08-14

**Authors:** Guisheng Zhou, Junzhi Zhang, Hongli Guo, Xiaochao Hu, Yingzhuo Wang, Kunqun Shi, Tongtong Liu, Shengyan Yin, Huanhuan Liu, Chunling Liu, Shijia Liu

**Affiliations:** ^1^ Affiliated Hospital of Nanjing University of Chinese Medicine Jiangsu Province Hospital of Chinese Medicine 210029, Nanjing, China; ^2^ Jiangsu Collaborative Innovation Center of Chinese Medicinal Resources Industrialization and Jiangsu Key Laboratory for High Technology Research of TCM Formulae Nanjing University of Chinese Medicine 210023, Nanjing, China; ^3^ College of the First Clinical Medicine Nanjing University of Chinese Medicine 210023, Nanjing, China; ^4^ Pharmaceutical Sciences Research Center Department of Pharmacy Children's Hospital of Nanjing Medical University 210008, Nanjing, China

**Keywords:** biomarker, heart failure, metabolite profile, serum metabolomics, UPLC/Q-TOF-MS

## Abstract

Detection of biomarkers was extremely important for the early diagnosis, prognosis, and therapy optimization of diseases. The purpose of this study was to investigate the differences in serum metabolites between patients with heart failure (HF) and healthy control (HC) and to diagnose HF qualitatively. In this study, serum samples from 83 patients with HF and 35 HCs were used as the research subjects for untargeted metabolomic analysis using ultraperformance liquid chromatography combined with quadrupole-time of flight mass spectrometry (UPLC-QTOF/MS) technology. Potential biomarkers were screened and validated using the orthogonal partial least squares discriminant analysis (OPLS-DA), random forest (RF), binary logistic regression (BLR), and receiver operating characteristic (ROC) analysis. The results indicated that a total of 43 metabolites were considered as differentially expressed metabolites (DEMs). Among these DEMs, glycodeoxycholate was identified as a specific biomarker of HF. A ROC curve analysis for HC versus HF discrimination showed an area under the ROC curve (AUC) of 0.9853 (95% CI: 0.9859–1.0000), a sensitivity of 95%, and a specificity of 100%. Hence, glycodeoxycholate might serve as a potential biomarker for HF. Furthermore, the amino acid metabolism was screened as the most significantly altered pathway in patients with HF. By identifying serum biomarkers and analyzing metabolic pathways, our study provided opportunities to enhance the understanding of the pathogenesis and early diagnosis of HF.

## 1. Introduction

Heart failure (HF) was a serious disorder of cardiac systolic and/or diastolic function, impaired ventricular filling and/or ejection, and insufficient cardiac output due to various structural or functional diseases of the heart. Pathophysiological syndrome resulting in a series of clinical manifestations (such as dyspnea, varying degrees of physical activity limitation, and fluid retention) was also the terminal stage of the development of various cardiac diseases (such as myocardial infarction, hypertension, aortic stenosis, valvar insufficiency, and arrhythmia) [1].

The American College of Cardiology/American Heart Association (ACC/AHA) define HF as “a complex clinical syndrome caused by any structural or functional disorder of ventricular filling or blood ejection” [2]. Diagnosis and assessment of HF relied on history, physical examination, and chest X-rays. As well as diagnostic and prognostic biomarkers of HF such as b-type natriuretic peptide (BNP) and n-terminal proBNP (NT-proBNP) [3, 4], among which, detection of biomarkers could provide disease information conveniently and quickly [5]. The detection of HF-related biomarkers became an available tool for rapid diagnosis of HF and provided important reference information for risk stratification and prognosis of patients with HF.

Metabolomics was often used as a tool for biomarker discovery through comprehensive analysis of small molecule metabolites in cells, tissues, and body fluids [6, 7]. Metabolomics was the research object of all metabolites in the body, and these metabolites were generated by the reaction of endogenous substances in the body, which was the ultimate embodiment of the overall function or state of the biological system and the comprehensive effect of multiple factors. It was widely used to identify biomarkers of HF in recent years [8, 9].

Due to the complexity of HF, there might be a large number of metabolites in the myocardium. Therefore, a comprehensive metabonomic analysis could reflect the pathological changes in the occurrence and development of HF. The metabolites were analyzed by metabolomic technologies and involved in metabolic intermediates such as substrates and products of carbohydrate, amino acids, fatty acids, and ketones metabolism, which participated in cell and organism homeostasis, and were used to explore HF-related systemic metabolic dysfunction. Given that metabolic changes were expected to occur in HF patients, coupled with the important role played by changes in metabolites in regulating comorbidities, we performed an extended clinical metabolomics, to evaluate the related metabolic changes in HF patients [10, 11]. Clinical metabolomics was recently defined “as a new integrative biomedicine to discover the correlation and regulation between a large scale of metabolites measured and analyzed in liquid biopsies from patients with those patient phenomes and clinical phenotypes” [11]. The field of clinical metabolomics was also related to clinical phenomics. To date, clinical metabolomics has aided in determination of systemic metabolic mechanisms, as well as identification of diagnostic biomarkers and therapeutic targets [10, 11].

**Figure 1:**
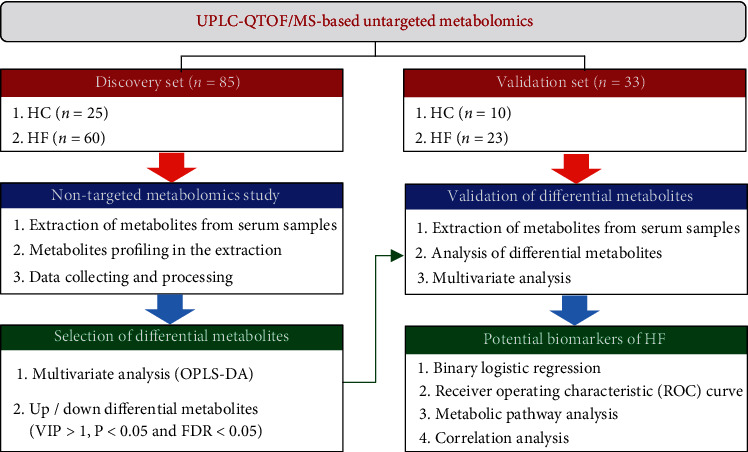
Workflow of this study.

Therefore, in this study, we collected getatable serum samples from patients with HF and healthy controls (HCs) and then performed clinical metabolomics to identify novel biomarkers for the diagnosis and management of HF. A flowchart of this study is presented in Figure 1.

## 2. Materials and Methods

### 2.1. Study Population

All procedures were approved by the Institutional Review Committee and Ethics Committee of the Affiliated Hospital of Nanjing University of Chinese Medicine (2019NL-081-02) and registered in the Chinese Clinical Trial Registry. This study complied with the Declaration of Helsinki. In this study, before collecting the subjects' serum samples, the subjects and their legal representatives were definitely required to carefully read the full contents of the informed consent, and all the questions from subjects and their legal representatives were detailedly answered by the researchers. Finally, in order to increase the awareness of the study, the participant and their corresponding legal representatives were also simultaneously required to sign the informed consent.

Clinical serum samples were collected from the Affiliated Hospital of Nanjing University of Chinese Medicine. A total of 118 samples were included, including 83 patients with HF and 35 HCs. Detailed clinical characteristics of the subjects are shown in Table 1. Detection of left ventricular ejection fraction (LVEF) was usually evaluated using a Siemens ACUSON SC2000 by two-dimensional echocardiographic testing hospital ultrasound systems. In the hospital clinical laboratory, the levels of NT-proBNP in blood samples were measured by the VIDAS automated enzyme-linked immunosorbent assay system with fluorescent immunostaining using the kit VIDAS PBN2. Blood samples were collected from volunteers who had fasted overnight via a venipuncture method and then processed and collected into blood separation gel tubes.

### 2.2. Sample Preparation and Analysis

Sample preparation was performed as follows. (1) The collected whole blood was centrifuged for 8 min at 4000 rpm to obtain serum. (2) An aliquot of serum (25 *μ*L) was added to 100 *μ*L of isopropyl alcohol, followed by vortex-mixing for 3 min to obtain the mixture. (3) The mixture was placed at 4°C for 2 h and centrifuged at 12,000 rpm for 15 min in a refrigerated centrifuge (at 4°C). (4) The supernatant was transferred into a sample bottle and stored at 4°C for MS analysis of metabolomics. A quality control (QC) sample was created by mixing equal volumes of each test sample (10 *μ*L) and treated as described above. During the analysis run, the pooled QC samples were injected for every 10 samples for repeatability [12].

### 2.3. Metabolomic Analysis Using UPLC-QTOF-MS

UPLC-QTOF-MS analyses were performed using a TripleTOF™ 5600+ high-resolution mass spectrometer (AB SCIEX, United States) and an ExionLC™ high-performance liquid chromatography system (AB SCIEX, United States). Waters HSS T3 column (100 × 2.1 mm, 1.7 *μ*m) was used for separation of small polar metabolites in positive electrospray ionization (ESI^+^) mode. The mobile phase was composed of 0.1% formic acid in water (A) and acetonitrile (B) using a gradient elution of 1% B at 0 min, 1% B at 1.5 min, 99% B at 13 min, and 99% B at 16.5 min. The flow rate, injection volume, column temperature, and sample manager were set at 0.3 mL/min, 2 *μ*L, 40°C, and 4°C, respectively. Detailed data are provided in Table S1.

The Acquity UPLC BEH Amide column (100 × 2.1 mm, 1.7 *μ*m) was used in the negative electrospray ionization (ESI^−^) mode for the analysis of polar metabolites. The column temperature was maintained at 40°C, the temperature of the sample plate was maintained at 4°C, and the sample volume was 2 *μ*L. The mobile phase consisting of 5 mmol/L ammonium acetate and 0.5% acetic acid in water (A) and acetonitrile (B) was delivered at a flow rate of 0.3 mL/min using a gradient program as follows: 95% (B) from 0 to 1 min, 95%–65% (B) from 1 to 14 min, 65%–40% (B) from 14 to 16 min, 40% (B) from 16 to 18 min, 40%–95% (B) from 18 to 19 min, and 95% (B) from 19 to 23 min. Detailed data are provided in Table S2.

The information-dependent acquisition (IDA) was used to acquire MS/MS spectra for ions matching the IDA criteria. The TOF-MS was acquired scanning a mass range from *m/z* 50–1000 followed by product ion scanning from *m/z* 50–1000 with 200 and 0.05 ms accumulation times, respectively. Spray voltage for ESI^+^ and ESI^−^ was set at 5500 and 4500 V, respectively. The other operating MS parameters were used, as follows: nebulizer gas (GS1), 50 Pa; auxiliary gas (Gas 2), 50 Pa; curtain gas (CG), 40 Pa; ion source temperature, 500°C; decluttering potential (DP), 80 V; and the collision energy (CE), 35 ± 15 V.

### 2.4. Data Analysis

Data processing raw files were converted into mzXML format using the MSconvert module of ProteoWizard software. XCMS, MetDNA, and MasterView software were used for identification of compounds according to retention time, accurate mass number, secondary mass spectrometry, and other information. The peak area of qualitative compounds was quantified by MultiQuant software, and then, the differences of various compounds between the two groups were statistically analyzed by MetaboAnalyst 5.0 online software.

Principal component analysis (PCA) was firstly performed to visualize the general grouping trend of all samples on the score plot, and the quality of data was evaluated using the tight clustering of QC samples. The processed data were further exported into the SIMCA software (Version 14.1; Umetrics) for orthogonal partial least squares discriminant analysis (OPLS-DA). The variables were selected based on variable importance in the projection (VIP) values in OPLS-DA models, and VIP > 1.0 was considered as a significant difference. A *t*-test was applied for the statistical analysis, to determine the statistical significance of differences in the metabolites (*p* < 0.05). False discovery rate (FDR) was corrected for using the Benjamini–Hochberg method. Metabolites with the statistical thresholds of VIP > 1, *p* < 0.05, and FDR < 0.05 were considered to be differentially expressed metabolites (DEMs). Random forest (RF) and binary logistic regression (BLR) models were applied to find the significantly altered metabolites. The clinical diagnostic efficiency of the regression analysis was evaluated by performing a receiver operating characteristic (ROC) curve analysis, and the area under the ROC curve (AUC) was utilized to identify and confirm the significantly altered metabolites as potential diagnostic biomarkers for HF. The Pearson correlation coefficient was calculated to determine the correlation between DEMs and some clinical parameters. MetaboAnalyst 5.0 software was used to analyze the pathways of DEMs. Pathway and metabolite accumulation analyses were applied to identify pathways most related to HF.

The SPSS25.0 (International Business Machines Corporation, Armonk, NY, United States) was utilized to analyze data from ROC and BLR. RF analysis was performed on MetaboAnalyst 5.0. Subsequently, a heat map was generated using the R software. OPLS-DA was performed and coded using the SIMCA 13.0 software (Umetrics, Sweden).

## 3. Results

### 3.1. Basic Characteristics of the Participants

In this study, 85 subjects (60 with HF and 23 HCs) were assigned to the discovery set to evaluate biomarkers, and 33 subjects (23 with HF and 10 HCs) were assigned to the validation set to test candidate biomarkers. A summary of clinical and demographic characteristics of subjects in both groups is shown in Table 1. HF was about equally likely to develop in men and women, so we did not consider gender differences between the groups. It was found that no significant differences were observed in the course of disease of HF subjects across both study sets. However, there were significant differences between HF and HC groups with regard to LVEF and NT-proBNP.

### 3.2. Multivariate Statistical Analysis

**Figure 2:**
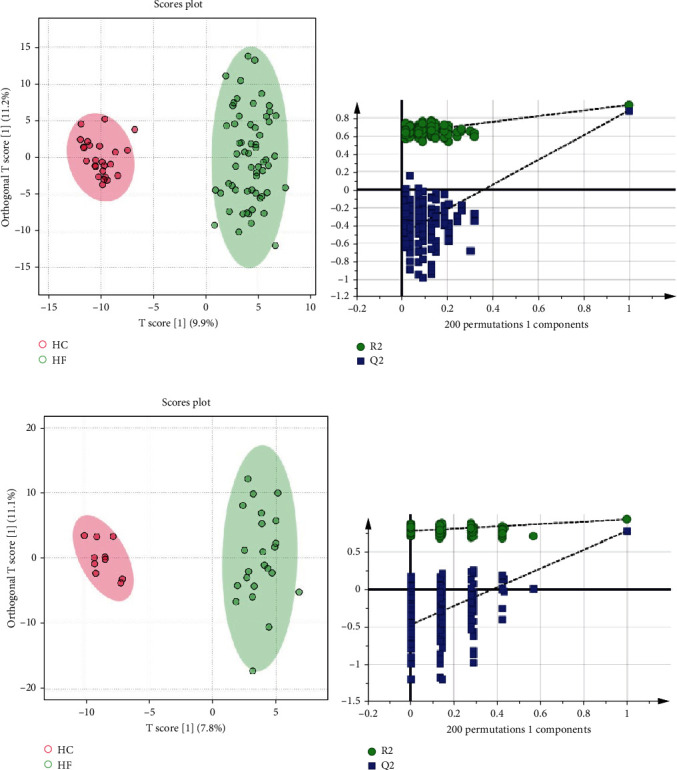
Identification of potential metabolic biomarkers for the diagnosis of HF in the discovery set and validation set. (a) OPLS-DA score plot based on HC and HF groups in the discovery set. (b) Two hundred permutation tests of OPLS-DA model in the discovery set. (c) OPLS-DA score plot based on HC and HF groups in the validation set. (d) Two hundred permutation tests of OPLS-DA model in the discovery set.

In the metabolomic analysis, 453 metabolites were detected in serum samples from the discovery set. Subsequently, all the identified metabolites were subjected to OPLS-DA model. The results of OPLS-DA analysis showed that clear separation was observed between patients with HF and HC (Figure 2(a)). Furthermore, 200-permutation test was further applied to validate the OPLS-DA model, confirming the robustness of the model (Figure 2(b)). The result of permutation test showed that OPLS-DA model was robust in reflecting the differences between HF and HC groups, and overfitting was not a problem in this model. Subsequently, 158 metabolites with significant changes were screened from the discovery set based on the statistical significance criteria of VIP > 1, *p* < 0.05, and FDR < 0.05.

### 3.3. Identification and Performance of Potential Diagnostic Biomarkers

The screening of biomarkers was an important part of omics research. The misidentification of biomarkers would lead to the misdiagnosis of diseases, the inaccuracy of the prognosis of diseases, and the efficacy of drugs. Therefore, a reliable selection process appeared particularly important for screening the potential biomarkers. In the present study, we divided the selection process into the following three steps: (1) DEMs were sorted in descending order according to VIP and *p* values and lists established; (2) DEMs should have consistent trends in both discovery and validation set; and (3) the representative metabolites were further screened by RF model, BLR analysis, and ROC analysis.

In this study, the untargeted metabolomic identified 158 DEMs between HF and HC groups in the discovery set. The serum samples from the validation set were employed to further evaluate the reliability of 158 DEMs and screened key metabolites as potential diagnostic biomarkers for distinguishing patients with HF from HCs. The score plot of OPLS-DA for patients with HF versus HCs showed a distinct separation between the two groups (Figure 2(c)) in the validation set, as in agreement with the result of OPLS-DA in the discovery set. Moreover, model validation by permutation test confirmed the reliability of OPLS-DA model in explaining and predicting the variation between the two groups (HF and HC) in the validation set (Figure 2(d)). To validate the findings in discovery set, 158 DEMs listed above were further verified and analyzed in the validation set using the same analytical procedures as the discovery set. Notably, 43 of 158 metabolites showed significant differences between the two comparisons (HF and HC) and presented consistent trends in both discovery and validation sets.

**Figure 3:**
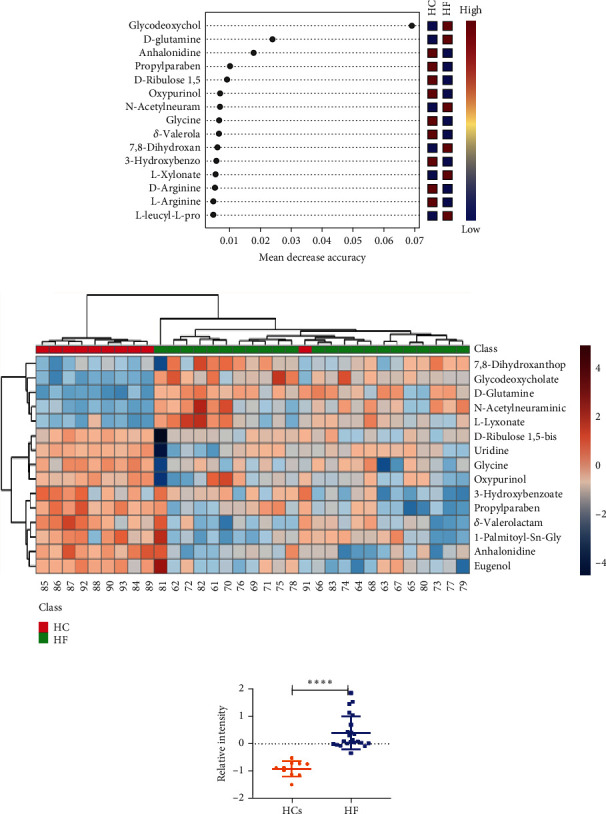
(a) Mean decrease accuracy values of the metabolic biomarkers used for random forest classification. (b) Heat map of 24 differential metabolites. (c) Pathway analysis of the differentially altered metabolites identified in patients with HF.

Compared with the other tools, RF model was an excellent and effective tool for screening biomarkers in metabolomics. As shown in Figure 3(a), 15 of 43 DEMs were screened by RF model, and glycodeoxycholate, D-glutamine, and anhalonidine were ranked as the Top 3 DEMs by the values of mean decrease accuracy (MDA). In order to further visualize the distribution of DEMs in each comparison group from validation set, hierarchical clustering algorithm (HCA) was used to perform cluster analysis on the DEMs, and the data was displayed in the form of heat map (Figure 3(b)). As shown in Figure 3(b), the brown color represented higher relative abundance, whereas the blue color represented lower relative abundance. MetaboAnalyst 5.0 software was used to analyze the pathway affected by the 15 DEMs, and all annotated metabolites were mapped into biochemical pathways to promote a mechanistic interpretation (Figure 3(c) and Table 2). There were four significantly different pathways associated with HF including phenylalanine, tyrosine and tryptophan biosynthesis, glycine, serine and threonine metabolism, phenylalanine metabolism, and ketone body synthesis and degradation. Fifteen DEMs screened by RF and HCA were further subjected to a BLR analysis in the validation set. Using a forward stepwise optimization algorithm (Wald), glycodeoxycholate was identified as a reliable diagnostic potential DEM in the regression model. The serum relative intensity of glycodeoxycholate is shown in Figure 3(d). The result indicated that glycodeoxycholate was overexpressed in HF but significantly reduced in HC. Glycodeoxycholic acid was involved in the metabolism of primary biliary oleic acid, which was confirmed to be related to a variety of diseases, including liver fibrosis [13] and asthma [14].

**Figure 4:**
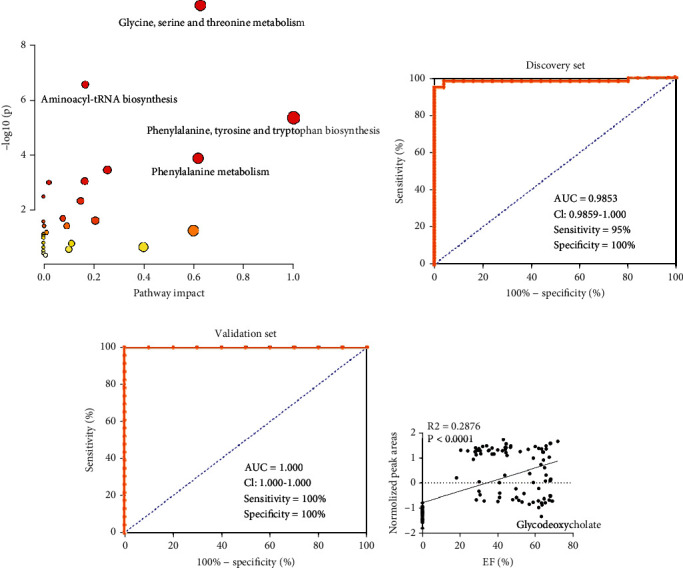
(a) ROC curve analysis of glycodeoxycholate in the discovery set. (b) ROC curve analysis of glycodeoxycholate in the validation set. (c) Correlation analysis between the glycodeoxycholate and LVEF. (d) Scatter plot of the relative intensity of glycodeoxycholate.

The AUC of the potential biomarker was calculated by ROC curves, which were frequently used in biomedical informatics research to evaluate the classification and prediction model decision support, diagnosis, and prognosis. In this study, the diagnostic potential of glycodeoxycholate was subsequently evaluated by ROC analysis. The AUC, 95% confidence interval (95% CI), sensitivity, and specificity for ROC curves calculated at optimal cutoff with Youden's index are displayed in Figure 4(a). The AUC value of potential biomarkers in this study was 0.9853 in the discovery set and 1.000 in the validation set (Figure 4(b)). Finally, we analyzed the biological relevance between glycodeoxycholate as a potential biomarker and LVEF to ascertain the ideal biomarker for detecting GLL. The results showed that glycodeoxycholate positively correlated with LVEF (Figure 4(c)). These results further indicated that glycoside deoxycholate could be used as a diagnostic biomarker for HF.

## 4. Discussion

HF was a common and critical disease of the cardiovascular system. The symptoms and signs of patients with HF were often nonspecific, and it was difficult to make an accurate diagnosis only by clinical manifestations. The emergence of HF biomarkers is related to improve the discrimination of HF [15]. Metabonomics, a branch of system biology, was a new discipline following genomics, transcriptomics, and proteomics and was the omics closest to phenotype [16]. One focus of metabonomic research was to discover metabolites associated with disease and environmental exposure, thus providing reference for the diagnosis and mechanism of disease research. The number of metabolites was much smaller than the number of genes and proteins, making metabolomics relatively convenient to study. Currently, the technical means of metabonomics included gas chromatography-mass spectrometry (GC-MS) [17–19], LC-MS [20], capillary electrophoretic-mass spectrometry (CE-MS) [21], and nuclear magnetic resonance (NMR) [22, 23]. Among them, LC-MS was widely used due to its characteristics of high sensitivity, high selectivity, and good metabolite coverage. Metabolic impairment was a key factor in the pathogenesis and progression of HF [24]. The previous reports revealed that circulating amino acids and long-chain acylcarnitine levels are strongly associated with the progression of HF. These metabolites contributed to the early differentiation of cardiovascular disease such as coronary heart disease (CHD), valvular heart disease (VHD), and dilated cardiomyopathy (DCM). Moreover, these amino acids and long-chain acyl carnitines provided new diagnostic targets or therapeutic interventions [25]. Hunter et al. used the targeted metabolomics and enzymatic method to quantify 63 metabolites in fasting serum samples and identified novel circulating metabolites reflecting impaired or dysregulated fatty acid oxidation that are independently associated with HF [26]. Using untargeted metabolomics to identify and validate changes in blood metabolite profiles, the other study showed significant dysregulation of phospholipids in chronic HF [27]. LC-MS/MS was used for therapeutic monitoring of serum digoxin in patients with HF [28]. Additionally, several dysregulated small molecules were also successfully identified in HF patients [29]. LC-MS/MS technology was used to analyze the changes of metabolites in serum samples of elderly patients with congestive HF, and serum and fecal biomarkers could be used for HF screening in elderly patients [30]. Although studies shown that many biomarkers could supplement the prediction of HF, there were no new biomarkers for clinical use. Our study presented the preliminary comparative metabolomic method to discovery a panel of serum biomarkers in HF and generated a robust set of candidate biomarkers for the diagnosis of HF. Remarkably, multivariate analysis revealed significant differences in metabolomic profiles between patients with HF and HC, revealing an evident impact of disease state on the levels of serum metabolites. As a result, 453 metabolites were identified, of which 43 metabolites were considered as DEMs. In HF patients, most identified DEMs presented an important role in amino acid metabolism, fatty acid metabolism, inflammatory response, etc. Hence, current data strongly supported the idea that metabolic disorders were the primary culprit in HF pathogenesis. Based on the clinical metabonomic data, glycodeoxycholate was screened and confirmed as a potential biomarker for distinguishing HF.

According to relevant data analysis [31], the 3-year survival rate of patients with HF was only about 68%, while the 5-year survival rate drops to about 50%. Some scholars believed that the 5-year survival rate of patients with HF was even comparable to that of patients with malignant tumor (about 34%). In this study, based on the serum metabolomic analysis of HF patients and HC, we screened and obtained a variety of differential metabolites, then established a diagnostic model by BLR analysis, and obtained a potential biomarker (glycodeoxycholate) for distinguishing HF. Glycodeoxycholate was bile salt formed in the liver by conjugation of deoxycholate with glycine, usually as the sodium salt. Glycodeoxycholate acted as a detergent to solubilize fats for absorption, and it usually used as cholagogue and choleretic. When diagnosing HF, the AUC of ROC curve reached 0.9853, indicating that it presented good diagnostic efficacy and could provide reference for the diagnosis and treatment of HF.

There were currently 15 different metabolites mainly involved in amino acid metabolism pathways. Amino acid played a vital role in the metabolism of heart, because they were in protein synthesis and energy substrate metabolism necessary intermediary metabolism [32]. Several clinical trials showed that complement amino acid could improve exercise tolerance in patients with HF and in some cases [33].

As with any clinical metabolomic analysis, there were some limitations to our study. The current implementation was limited in sample cohort size from one center. Also, the sample number is relatively small, especially for metabolomic analysis in the validation stage. We expected to improve in both sensitivity and specificity with inclusion of more samples from multicenters in the future studies. It should be noted that we are currently designing and preparing to implement validation of our results through large sample sizes, multicenter, parallel group, and direct comparative studies. Moreover, the metabolomics itself showed some limitations. A single instrument to detect all metabolites in a single analysis was not available, and the data presented here suffered from incomplete metabolite annotation that left several interesting pathways underexplored. In the further studies, the multiomics technology could be employed to identify more potential specific biomarkers by analyzing multiple biological matrices including serum, urine, feces, and saliva.

## 5. Conclusions

In summary, the clinical metabolomics method was applied to comprehensively identify perturbations in serum metabolites in a cohort of patients with HF. Our results revealed disorders in a number of DEM changes associated with important biological processes, including phenylalanine, tyrosine and tryptophan biosynthesis, glycine, serine and threonine metabolism, phenylalanine metabolism, and ketone body synthesis and degradation in HF. Notably, glycodeoxycholate was screened and validated as a potential diagnostic biomarker for the diagnosis of HF by RF, BLR, and ROC analyses. These findings provided new insights to improve the diagnosis and treatment of HF and further enhanced our understanding of HF pathophysiology.

## Figures and Tables

**Table 1:** The clinical features of the subjects.

**Parameter**	**Discovery set (** **n** = 85**)**		**Validation set (** **n** = 33**)**	
	**HF**	**HC**	**HF**	**HC**
Number (*n*)	60	25	23	10
Male (*n*)	35	9	16	3
Female (*n*)	25	16	7	7
LVEF(%) (*M* ± SD)	47.41 ± 014	—	55.02 ± 0.28	—
NT-proBNP (*n* ≥ 300 pg/mL)	1793.3 ± 37.5	—	1848.2 ± 41.6	—
cTnL (*n* ≥ 0.5 ng/mL)	0.11 ± 0.22	—	0.07 ± 0.08	—
AST (15–40 U/I)	23.14 ± 5.01	16.2 ± 5.80	21.41 ± 5.08	13.4 ± 7.24
ALT (9–50 U/L)	21.47 ± 9.14	16.4 ± 2.71	24.24 ± 5.08	15.56 ± 2.96
BUN (2.5–7.0 *μ*M/L)	5.89 ± 1.69	5.04 ± 1.35	5.51 ± 1.39	4.47 ± 1.06
Cr (45–133 *μ*M/L)	72.66 ± 12.53	71.9 ± 16.31	71.9 ± 11.02	70.02 ± 16.39

Abbreviations: ALT: alanine aminotransferase; AST: aspartate transaminase; BUN: blood urea nitrogen; Cr: Creatinine; cTnl: cardiac troponin l; DM: diabetes mellitus; HL: hyperlipidemia; HT: hypertension; LVEF: left ventricular ejection fraction; NA: not available; NTproBNP: N-terminal-pro-B-type natriuretic peptide; SD: standard deviation.

^a^Class of New York Heart Association functional classifications.

^b^Values are mean ± SD.

**Table 2:** DEMs identified between patients with HF and HC.

**Metabolite**	**Discovery set**				**Validation set**			
	**p** ** value** ^a^	**FDR** ^b^	**VIP** ^c^	**FC** ^d^	**p** ** value**	**FDR**	**VIP**	**FC**
Glycodeoxycholate	< 0.001	< 0.001	2.32407	364.61	< 0.001	< 0.001	2.4983	20.236
D-Glutamine	< 0.001	< 0.001	1.2019	1.7376	< 0.001	< 0.001	2.54803	2.9254
Anhalonidine	< 0.001	< 0.001	1.59768	0.59687	< 0.001	< 0.001	2.25459	0.61403
Propylparaben	< 0.001	< 0.001	1.63576	0.52666	< 0.001	< 0.001	1.97718	0.52992
D-Ribulose 1,5-bisphosphate	< 0.001	< 0.001	1.50855	0.66194	< 0.001	< 0.001	1.21421	0.60113
Oxypurinol	< 0.001	< 0.001	1.58551	0.53493	< 0.001	< 0.001	1.27317	0.69278
N-Acetylneuraminic acid	< 0.001	< 0.001	1.40426	3.7164	< 0.001	< 0.001	2.315	5.4645
Glycine	< 0.001	< 0.001	1.183	0.73774	< 0.001	< 0.001	1.45637	0.63467
delta-Valerolactam	< 0.001	< 0.001	1.48184	0.56017	< 0.001	< 0.001	2.04797	0.58129
7,8-Dihydroxanthopterin	< 0.001	< 0.001	1.86708	2.9846	< 0.001	< 0.001	1.66909	2.7554
3-Hydroxybenzoate	< 0.001	< 0.001	1.39384	0.43224	< 0.001	< 0.001	1.59786	0.51962
L-Xylonate	< 0.001	< 0.001	1.25809	1.8564	< 0.001	< 0.001	1.97149	1.733
D-Arginine	< 0.001	< 0.001	1.16534	0.75364	< 0.001	< 0.001	2.14929	0.57645
L-Arginine	< 0.001	< 0.001	1.16546	0.75358	< 0.001	< 0.001	2.1505	0.57635
L-leucyl-L-proline	< 0.001	< 0.001	1.62239	2.7065	< 0.001	< 0.001	2.31127	2.2743

^a^ *p* values were obtained from one-way ANOVA.

^b^The value of FDR was obtained from the adjusted *p* value calculated using MetaboAnalyst 5.0 software.

^c^VIP was obtained from the OPLS-DA model with a threshold of 1.0.

^d^The value of FC was obtained by comparing metabolites in patients with HF with HC.

## Data Availability

The data used or analyzed during the current study are available from the corresponding author upon reasonable request. The data set has been uploaded to MetaboLights and is currently under review. It will be made publicly available for public access once approved (URL: http://www.ebi.ac.uk/metabolights/MTBLS10032), and the accession number is MTBLS9586.

## Supporting Information

Supporting Information Additional supporting information can be found online in the Supporting Information section. The table shows the different elution times and proportions of phases A and B in positive and negative ion mode liquid chromatography.
