# Supplementary material for: Discovery and Validation of Potential Serum Biomarkers for Heart Failure by Untargeted Metabolomics
Source: Cardiovasc Ther. 2024 Aug 14;2024:7004371. doi: 10.1155/2024/7004371 (PMC11338663; doi:10.1155/2024/7004371)
Supplement: Supporting Information — Additional supporting information can be found online in the Supporting Information section. The table shows the different elution times and proportions of phases A and B in positive and negative ion mode liquid chromatography. [file 7004371.f1.docx]

**STable 1** Positive ion mode liquid chromatography gradient elution schedule

| **Time（min）** | **liquid-phase A（v%）** | **liquid-phase B（v%）** |
| --- | --- | --- |
| 0.01 | 99 | 1 |
| 1.5 | 99 | 1 |
| 13 | 1 | 99 |
| 16.5 | 1 | 99 |
| 16.6 | 99 | 1 |
| 20 | 99 | 1 |

This is the ratio of different elution times and phases A and B in positive ion mode liquid chromatography

**STable 2** Negative ion mode liquid chromatography gradient elution schedule

| **Time（min）** | **liquid-phase A（v%）** | **liquid-phase（v%）** |
| --- | --- | --- |
| 1 | 5 | 95 |
| 14 | 35 | 65 |
| 16 | 60 | 40 |
| 18 | 60 | 40 |
| 18.1 | 5 | 95 |
| 23 | 5 | 95 |

This is the ratio of different elution times and phases A and B in negative ion mode liquid chromatography
